# Prevalence of Asthma, Asthma Attacks, and Emergency Department Visits for Asthma Among Working Adults — National Health Interview Survey, 2011–2016

**DOI:** 10.15585/mmwr.mm6713a1

**Published:** 2018-04-06

**Authors:** Jacek M. Mazurek, Girija Syamlal

**Affiliations:** 1 Respiratory Health Division, National Institute for Occupational Safety and Health, CDC.

In 2010, an estimated 8.2% of U.S. adults had current asthma, and among these persons, 49.1% had had an asthma attack during the past year (*1*). Workplace exposures can cause asthma in a previously healthy worker or can trigger asthma exacerbations in workers with current asthma* (*2*). To assess the industry- and occupation-specific prevalence of current asthma, asthma attacks, and asthma-related emergency department (ED) visits among working adults, CDC analyzed 2011–2016 National Health Interview Survey (NHIS) data for participants aged ≥18 years who, at the time of the survey, were employed at some time during the 12 months preceding the interview. During 2011–2016, 6.8% of adults (11 million) employed at any time in the past 12 months had current asthma; among those, 44.7% experienced an asthma attack, and 9.9% had an asthma-related ED visit in the previous year. Current asthma prevalence was highest among workers in the health care and social assistance industry (8.8%) and in health care support occupations (8.8%). The increased prevalence of current asthma, asthma attacks, and asthma-related ED visits in certain industries and occupations might indicate increased risks for these health outcomes associated with workplace exposures. These findings might assist health care and public health professionals in identifying workers in industries and occupations with a high prevalence of current asthma, asthma attacks, and asthma-related ED visits who should be evaluated for possible work-related asthma. Guidelines intended to promote effective management of work-related asthma are available (*2*,*3*). 

The NHIS is an annual survey that collects health information from a nationally representative sample of the noninstitutionalized U.S. civilian population through personal interviews.^†^ Survey participants were considered to be working in the last 12 months if they reported having a job or business at any time during the past 12 months.^§^ For analyses, information on respondents’ current industry (21 major groups/79 detailed industries) and occupation (23 major groups/94 detailed occupations) were used.^¶^ Participants who had ever been told by a doctor or other health professional that they had asthma and reported that they still have asthma were considered to have current asthma. Persons with at least one asthma attack in the past year, or at least one asthma-related ED visit in the past year were identified by affirmative responses to questions “During the past 12 months, have you had an episode of asthma or an asthma attack?” and “During the past 12 months, have you had to visit an emergency room or urgent care center because of asthma?,” respectively.

Data were weighted to produce nationally representative estimates using sample weights, and variance estimates were calculated to account for the clustered survey design. Estimates with a relative standard error (standard error of the estimate divided by the estimate) ≥30% were not reported. The Rao-Scott chi-square test was used to determine statistically significant differences (p<0.05) between groups. Data were analyzed using statistical software.

During 2011–2016, an estimated (annual average) 160.7 million adults were working at any time during the past 12 months (Table 1), 6.8% (11.0 million) of whom had current asthma. Current asthma prevalence was highest among workers aged 18–24 years (8.5%), females (8.9%), non-Hispanic blacks (8.2%), those with higher than a high school education (7.2%), those categorized as “poor”** (8.7%), those having health insurance (7.1%), and those living in the Northeast (7.6%).

**TABLE 1:** Current asthma* prevalence and proportion of adults working at any time in the past 12 months^†^ with current asthma who had at least one asthma attack^§^ or emergency department (ED) visit for asthma^¶^ in the past 12 months (annual average), by selected characteristics — National Health Interview Survey, 2011–2016

Characteristic	Workers (x 1,000)**	Current asthma			Proportion of persons with current asthma who had ≥1 asthma attack in past 12 months		Proportion of persons with current asthma who had ≥1 asthma ED visit in past 12 months	
		No. (x 1,000)**	% (95% CI)	p-value	% (95% CI)	p-value	% (95% CI)	p-value
**Age group (yrs)**								
18–24	22,005	1,858	8.5 (7.8–9.1)	<0.001	36.8 (33.3–40.3)	<0.001	10.5 (8.0–13.0)	0.30
25–44	68,651	4,529	6.6 (6.3–6.9)		46.2 (44.1–48.4)		10.4 (9.2–11.7)	
45–64	60,927	4,072	6.7 (6.4–7.0)		47.4 (45.2–49.5)		9.3 (8.1–10.5)	
≥65	9,126	513	5.6 (5.0–6.2)		40.6 (34.4–45.8)		8.0 (5.5–10.5)	
**Sex**								
Men	84,415	4,181	5.0 (4.7–5.2)	<0.001	39.0 (36.6–41.4)	<0.001	7.0 (5.9–8.2)	<0.001
Women	76,294	6,791	8.9 (8.6–9.2)		48.4 (46.7–50.1)		11.7 (10.6–12.8)	
**Race/Ethnicity**								
Hispanic	25,359	1,272	5.0 (4.7–5.4)	<0.001	44.9 (41.1–48.7)	0.51	15.7 (12.7–18.7)	<0.001
White, non-Hispanic	106,291	7,667	7.2 (7.0–7.5)		45.4 (43.7–47.1)		7.6 (6.7–8.5)	
Black, non-Hispanic	18,770	1,542	8.2 (7.7–8.8)		42.5 (38.9–46.0)		17.6 (14.9–20.3)	
Other	10,289	490	4.8 (4.2–5.4)		42.9 (37.0–48.9)		7.1 (3.9–10.3)	
**Education level**								
≤High school	52,305	3,255	6.2 (5.9–6.5)	<0.001	43.8 (41.3–46.3)	0.62	13.3 (11.6–15.0)	<0.001
>High school	107,813	7,697	7.2 (6.9–7.4)		45.2 (43.6–46.9)		8.5 (7.6–9.4)	
Unknown	591	—^††^	—		—		—	
**Poverty index^§§^**								
Poor	14,335	1,251	8.7 (8.1–9.3)	<0.001	49.2 (45.5–52.9)	<0.001	17.0 (14.3–19.5)	<0.001
Near poor	23,012	1,617	7.0 (6.6–7.5)		44.3 (40.7–47.9)		12.8 (10.5–15.1)	
Not poor	114,200	7,544	6.6 (6.4–6.8)		44.2 (42.5–45.9)		7.9 (6.9–8.8)	
Unknown	9,163	560	6.2 (5.4–6.9)		44.5 (38.1–50.9)		13.5 (9.5–17.6)	
**Health insurance status**								
Not insured	24,577	1,344	5.5 (5.1–5.9)	<0.001	47.5 (43.3–51.6)	0.34	14.5 (12.0–17.1)	<0.001
Insured	135,328	9,576	7.1 (6.9–7.3)		44.5 (43.1–45.9)		9.3 (8.4–10.2)	
Unknown	804	52	6.4 (3.4–9.0)		—		—	
**Region**								
Northeast	28,621	2,182	7.6 (7.2–8.1)	<0.001	43.0 (40.5–45.4)	0.22	8.2 (6.5–9.9)	0.38
Midwest	37,804	2,679	7.1 (6.7–7.5)		43.7 (40.7–46.7)		8.9 (7.1–10.6)	
South	57,064	3,483	6.1 (5.8–6.4)		46.1 (43.5–48.8)		11.3 (9.7–12.9)	
West	37,220	2,628	7.1 (6.7–7.4)		45.7 (42.8–48.6)		10.6 (8.9–12.4)	
**Total**	**160,709**	**10,972**	**6.8 (6.7–7.0)**		**44.7 (43.3–46.1)**		**9.9 (9.1–10.7)**	

**Abbreviation:** CI = confidence interval.

* Defined as a “yes” response to the questions “Have you ever been told by a doctor or other health professional that you had asthma?” and “Do you still have asthma?”

^†^ Survey respondents who answered “yes” to the question “Did you have a job or business at any time in the past 12 months?”

^§^ Defined as a “yes” response to the question “During the past 12 months, have you had an episode of asthma or an asthma attack?”

^¶^ Defined as a “yes” response to the question “During the past 12 months, have you had to visit an emergency room or urgent care center because of asthma?”

** Weighted to provide national estimates.

^††^ Estimates suppressed because relative standard error for the estimate was ≥30%.

^§§^ Poverty index is based on family income and family size using the U.S. Census Bureau’s poverty thresholds for the previous calendar year. Persons who are categorized as “poor” have family incomes <100% of the poverty threshold, “near poor” have family incomes ≥100% to <200% of the poverty threshold, and “not poor” have family incomes ≥200% of the poverty threshold. ftp://ftp.cdc.gov/pub/Health_Statistics/NCHS/Dataset_Documentation/NHIS/2016/srvydesc.pdf, https://www.census.gov/data/tables/time-series/demo/income-poverty/historical-poverty-thresholds.html.

Among workers with current asthma, 44.7% (4.9 million) had at least one asthma attack, and 9.9% (1.1 million) had at least one asthma-related ED visit in the past 12 months (Table 1). The proportion of workers with current asthma who had at least one asthma attack was highest among workers aged 45–64 years (47.4%), females (48.4%), non-Hispanic whites (45.4%), those with higher than a high school education (45.2%), those categorized as poor (49.2%), those with no health insurance (47.5%), and those living in the South (46.1%). The proportion of workers with current asthma who had at least one asthma-related ED visit was highest among workers aged 18–24 years (10.5%), females (11.7%), non-Hispanic blacks (17.6%), those with less than high school education (13.3%), those categorized as poor (17.0%), those with no health insurance (14.5%), and those living in the South (11.3%).

By major industry, current asthma prevalence was highest among workers in the major industry groups of health care and social assistance (8.8%) followed by educational services (8.2%) (Table 2); these groups also had the first and second highest numbers of workers with asthma attacks (860,000 and 602,000, respectively) and asthma-related ED visits (212,000 and 102,000, respectively). The highest prevalence of asthma attacks was among workers with asthma in the transportation and warehousing (51.7%) industries, and the highest prevalence of asthma-related ED visits was among workers in retail trade (12.4%).

**TABLE 2:** Current asthma* prevalence and proportion of adults working at any time in the past 12 months^†^ with current asthma who had at least one asthma attack^§^ or emergency department (ED) visit for asthma^¶^ in the past 12 months (annual average), by industry** — National Health Interview Survey, 2011–2016

Industry	Workers (x 1,000)^††^	Current asthma		Proportion of persons with current asthma who had ≥1 asthma attack in past 12 months	Proportion of persons with current asthma who had ≥1 asthma ED visit in past 12 months
		No. (x 1,000)^††^	% (95% CI)	% (95%CI)	% (95% CI)
**Health care and social assistance**	21,270	1,878	8.8 (8.3–9.4)	45.8 (42.8–49.1)	11.3 (9.3–13.2)
Ambulatory health care services	8,135	710	8.7 (7.9–9.6)	43.2 (38.2–48.1)	10.5 (7.4–13.6)
Hospitals	6,693	551	8.2 (7.3–9.2)	51.7 (46.0–57.4)	10.1 (6.6–13.7)
Nursing and residential care facilities	2,978	262	8.8 (7.4–10.2)	45.2 (36.0–54.3)	13.8 (8.0–19.6)
Social assistance	3,463	355	10.3 (8.8–11.8)	42.9 (35.4–50.5)	12.3 (8.1–16.5)
**Education services**	15,237	1,243	8.2 (7.5–8.8)	48.4 (44.5–52.3)	8.2 (6.0–10.4)
**Arts, entertainment, and recreation**	3,569	287	8.1 (6.7–9.4)	50.0 (40.3–59.7)	—^§§^
Performing arts, spectator sports, and related	1,030	96	9.3 (6.5–12.1)	47.5 (31.0–64.0)	—
Museums, historical sites, and similar institutions	415	30	7.2 (3.6–10.7)	—	—
Amusement, gambling, and recreation	2,124	162	7.6 (5.9–9.4)	54.2 (41.7–66.6)	—
**Accommodation and food services**	11,233	864	7.7 (6.9–8.5)	40.1 (34.8–45.3)	11.0 (7.9–14.2)
Accommodation	1,737	151	8.7 (6.6–10.9)	51.3 (38.0–64.6)	—
Food services and drinking places	9,496	712	7.5 (6.7–8.4)	37.7 (32.2–43.1)	11.0 (7.5–14.5)
**Finance and insurance**	7,186	539	7.5 (6.6–8.5)	38.3 (32.4–44.2)	6.0 (3.1–9.0)
Monetary authorities –– central bank	2,122	140	6.6 (5.1–8.1)	31.9 (20.1–43.7)	—
Credit intermediation and related activities	1,197	83	6.9 (5.0–8.9)	46.0 (31.5–60.4)	—
Securities, commodity contracts, and other financial investments and related activities	1,163	80	6.9 (4.6–9.2)	34.8 (18.9–50.6)	—
Insurance carriers and related activities	2,704	237	8.8 (7.1–10.5)	40.6 (31.3–49.9)	—
**Retail trade**	16,714	1,247	7.5 (6.9–8.1)	46.4 (42.3–50.0)	12.4 (9.6–15.2)
Motor vehicle and parts dealers	1,807	114	6.3 (4.7–7.9)	44.4 (30.3–58.6)	—
Furniture and home furnishings stores	471	29	6.2 (3.1–9.4)	—	—
Electronics and appliance stores	549	65	11.9 (7.9–16.0)	37.8 (19.9–55.8)	—
Building material and garden equipment and supplies dealers	1,277	77	6.0 (4.1–8.0)	46.6 (30.1–63.1)	—
Food and beverage stores	3,241	215	6.7 (5.4–7.9)	45.8 (36.1–55.4)	14.1 (7.2–21.0)
Health and personal care stores	1,241	87	7.0 (4.9–9.1)	47.0 (31.7–62.4)	—
Gasoline stations	608	71	11.8 (7.8–15.7)	47.5 (30.0–65.0)	—
Clothing and clothing accessories stores	1,446	103	7.3 (5.3–9.2)	41.7 (29.0–54.5)	—
Sporting goods, camera, hobby, book and music stores	763	83	10.9 (7.8–13.9)	44.8 (28.8–60.7)	—
General merchandise stores	3,115	237	7.6 (6.1–9.2)	52.8 43.4–62.2)	13.1 (6.0–20.1)
Miscellaneous store retailers	1,140	102	9.0 (6.5–11.5)	50.4 (35.9–64.9)	—
Nonstore retailers and non-specified retail trade	1,057	64	6.0 (4.0–8.0)	45.1 (28.2–61.9)	—
**Public administration**	7,737	569	7.4 (6.5–8.2)	45.6 (39.1–52.1)	11.6 (6.4–16.7)
**Information**	3,438	228	6.6 (5.5–7.8)	48.8 (39.6–57.9)	13.1 (6.0–20.1)
Publishing industries (except internet)	702	48	6.9 (4.0–9.7)	51.2 (35.3–67.1)	—
Motion picture and sound recording industries	470	28	6.0 (3.5–8.5)	38.5 (17.1–60.0)	—
Broadcasting and telecommunications	1,742	106	6.1 (4.5–7.6)	49.1 (36.1–62.0)	—
Information services and data processing	524	45	8.7 (5.3–12.0)	51.8 (28.4–75.3)	—
**Professional, scientific, and technical services**	11,399	738	6.5 (5.8–7.2)	49.2 (44.2–54.1)	8.0 (5.2–10.7)
**Administrative & support and waste management & remediation services**	7,323	471	6.4 (5.6–7.2)	46.5 (40.4–52.6)	11.6 (8.0–15.3)
**Mining**	960	59	6.1 (3.9–8.3)	40.2 (21.6–58.8)	—
Oil and gas extraction	102	—	—	—	—
Mining (except oil and gas)	211	—	—	—	—
Support activities for mining	648	40	6.2 (3.4–9.0)	—	—
**Other services (except public administration)**	8,024	491	6.1 (5.4–6.9)	41.7 (35.3–48.2)	11.1 (7.4–14.9)
Repair and maintenance	2,287	121	5.3 (3.8–6.8)	33.6 (20.5–46.8)	—
Personal services (barber shops, beauty salons, nail salons, laundry, funeral homes and cemeteries)	2,219	117	5.3 (4.1–6.5)	37.4 (26.4–48.3)	16.5 (8.4–24.6)
Religious, grantmaking, civic, labor, professional, and similar organizations	2,486	162	6.5 (5.3–7.8)	44.1 (33.8–55.0)	—
Private households	1,032	91	8.9 (6.3–11.4)	53.2 (37.6–68.7)	22.9 (9.6–36.3)
**Utilities**	1,390	82	5.9 (4.3–7.6)	34.9 (20.3–49.4)	—
**Transportation and warehousing**	6,569	383	5.8 (5.0–6.7)	51.7 (43.4–60.0)	11.7 (7.7–15.6)
Transportation (including support activities for transportation)	4,544	245	5.4 (4.6–6.2)	55.1 (46.3–64.0)	14.9 (9.7–20.2)
Postal service, couriers, and messengers	1,460	108	7.4 (4.5–10.4)	44.4 (23.5–65.3)	—
Warehousing and storage	565	30	5.3 (2.8–7.8)	49.8 (25.1–74.5)	—
**Manufacturing**	16,067	860	5.4 (4.9–5.9)	40.0 (35.2–44.8)	6.7 (4.6–8.9)
Food manufacturing	1,954	104	5.3 (4.1–6.6)	33.1 (21.2–45.0)	—
Beverage and tobacco product manufacturing	301	—	—	—	—
Textile mills	98	—	—	—	—
Textile product mills	138	—	—	—	—
Apparel manufacturing	299	24	8.0 (3.2–12.7)	—	—
Leather and allied product manufacturing	28	—	—	—	—
Wood product manufacturing	447	34	7.7 (3.2–12.2)	57.3 (29.5–85.1)	—
Paper manufacturing	434	22	5.1 (2.2–7.9)	—	—
Printing and related support activities	613	36	5.9 (3.4–8.4)	46.2 (24.41–67.9)	—
Petroleum and coal products manufacturing	138	—	—	—	—
Chemical manufacturing	1,365	59	4.3 (3.1–5.6)	54.6 (31.5–77.6)	—
Plastics and rubber products manufacturing	549	20	3.6 (1.6–5.6)	56.7 (30.0–83.3)	—
Nonmetallic mineral product manufacturing	446	22	4.8 (2.1–7.6)	—	—
Primary metal manufacturing	572	43	7.5 (3.5–11.4)	—	—
Fabricated metal product manufacturing	1,228	55	4.5 (2.9–6.0)	34.0 (17.9–50.2)	—
Machinery manufacturing	1,437	80	5.6 (3.8–7.3)	47.2 (32.8–61.6)	—
Computer and electronic product manufacturing	1,316	70	5.3 (3.9–6.8)	44.1 (30.7–57.5)	—
Electrical equipment, appliance, and component manufacturing	496	32	6.5 (3.4–9.6)	—	—
Transportation equipment manufacturing	2,323	131	5.6 (4.4–6.9)	33.7 (23.1–44.3)	—
Furniture and related product manufacturing	483	26	5.5 (2.7–8.2)	—	—
Miscellaneous manufacturing	1,403	67	4.8 (3.3–6.3)	39.5 (23.9–55.1)	—
**Real estate and rental and leasing**	3,054	168	5.5 (4.3–6.7)	45.9 (34.9–56.9)	12.5 (4.4–20.5)
Real estate	2,643	142	5.4 (4.2–6.6)	48.7 (37.1–60.3)	14.0 (5.8–22.1)
Rental and leasing services	295	24	8.0 (3.0–11.6)	—	—
Lessors of nonfinancial intangible assets (except copyrighted works)	115	—	—	—	—
**Agriculture, forestry, fishing, and hunting**	2,358	123	5.2 (4.0–6.5)	38.8 (27.8–49.9)	—
Crop production	1,210	58	4.8 (3.2–6.3)	41.4 (26.8–56.1)	—
Animal production	680	44	6.5 (3.6–9.5)	—	—
Forestry and logging	171	—	—	—	—
Fishing, hunting, and trapping	68	—	—	—	—
Support activities for agriculture and forestry	228	—	—	—	—
**Wholesale trade**	3,898	192	4.9 (4.0–5.9)	36.7 (27.2–46.2)	—
Merchant wholesalers, durable goods	1,898	87	4.6 (3.1–5.9)	36.6 (21.3–51.9)	—
Merchant wholesalers, nondurable goods	1,963	104	5.3 (3.9–6.8)	37.3 (25.9–49.7)	—
Nonspecified wholesale trade	38	—	—	—	—
**Construction**	10,234	451	4.4 (3.8–5.0)	41.0 (33.8–48.1)	11.3 (6.8–15.9)
**Management of companies and enterprises**	111	—	—	—	—
**Armed forces**	360	—	—	—	—
Unknown	2,578	83	3.2 (2.4–4.1)	46.0 (32.3–59.8)	—
**Total**	**160,709**	**10,972**	6.8 **(6.7–7.0)**	**44.7 (43.3–46.1)**	**9.9 (9.1–10.7)**

**Abbreviation:** CI = confidence interval.

* Defined as a “yes” response to the questions “Have you ever been told by a doctor or other health professional that you had asthma?” and “Do you still have asthma?”

^†^ Survey respondents who answered “yes” to the question “Did you have a job or business at any time in the past 12 months?”

^§^ Defined as a “yes” response to the question “During the past 12 months, have you had an episode of asthma or an asthma attack?”

^¶^ Defined as a “yes” response to the question “During the past 12 months, have you had to visit an emergency room or urgent care center because of asthma?”

** Industry that employed sample adults were working in during the week prior to their interview. ftp://ftp.cdc.gov/pub/Health_Statistics/NCHS/Dataset_Documentation/NHIS/2014/srvydesc.pdf.

^††^ Weighted to provide national estimates.

^§§^ Estimates suppressed because relative standard error for the estimate was ≥30%.

By detailed industry sector, current asthma prevalence was highest among workers in electronics and appliance stores (11.9%) (Table 2). Among persons with current asthma, the highest asthma attack prevalence was among workers in wood products manufacturing (57.3%), followed by the plastics and rubber products manufacturing (56.7%), and the highest prevalence of asthma-related ED visits was among workers in private households (22.9%). The highest numbers of asthma attacks (307,000) and asthma-related ED visits (75,000) were among persons working in ambulatory health care services.

By major occupation group, current asthma prevalence was highest among workers in health care support (8.8%), followed by personal care and service (8.6%) occupations (Table 3). Among those with current asthma, the highest prevalence of asthma attacks was among workers in the education, training, and library (51.5%) major occupations; the highest prevalence of asthma-related ED visits was among workers in personal care and service (17.4%) occupations. The highest numbers of workers with asthma attacks (711,000) and asthma-related ED visits (137,000) were in the office and administrative support major occupation.

**TABLE 3:** Current asthma* prevalence and proportion of adults working at any time in the past 12 months^†^ with current asthma who had at least one asthma attack^§^ or emergency department (ED) visit for asthma^¶^ in the past 12 months (annual average), by occupation** — National Health Interview Survey, 2011–2016

Occupation	Workers (x 1,000)^††^	Current asthma		Proportion with current asthma and ≥1 asthma attack in past 12 months	Proportion with current asthma and ≥1 asthma ED visit in past 12 months
		No. (x 1,000)^††^	% (95% CI)	% (95% CI)	% (95% CI)	
**Health care support**	3,754	331	8.8 (7.6–10.0)	45.5 (38.4–52.6)	13.5 (9.1–18.0)	
Nursing, psychiatric, and home health aides	2,211	192	8.7 (7.1–10.2)	45.0 (35.9–54.2)	16.0 (9.7–22.3)	
Occupational and physical therapist assistants and aides	107	—^§§^	—	—	—	
Other health care support occupations	1,436	135	9.4 (7.3–11.5)	46.1 (35.2–57.1)	—	
**Personal care and service**	5,666	488	8.6 (7.5–9.7)	44.6 (38.8–50.5)	17.4 (12.9–21.8)	
Supervisors, personal care and service workers	167	14	8.1 (3.3–12.9)	—	—	
Animal care and service workers	285	26	9.0 (4.0–14.1)	—	—	
Entertainment attendants and related workers	310	—	—	—	—	
Funeral service workers	43	—	—	—	—	
Personal appearance workers	1,168	48	4.1 (2.6–5.6)	46.0 (28.1–63.8)	25.0 (10.3–39.7)	
Transportation, tourism, and lodging attendants	155	—	—	—	—	
Other personal care and service workers	3,539	364	10.3 (8.8–11.7)	44.8 (38.2–51.4)	16.2 (11.1–21.3)	
**Health care practitioners and technical**	8,752	754	8.6 (7.8–9.5)	49.7 (44.7–54.8)	8.9 (6.1–11.7)	
Health diagnosing and treating practitioners	5,991	534	8.9 (7.9–10.0)	49.4 (43.5–55.4)	8.6 (5.3–11.8)	
Health technologists and technicians	2,651	209	7.9 (6.4–9.3)	48.8 (39.1–58.5)	8.6 (3.8–13.4)	
Other health care practitioners and technical	110	—	—	—	—	
**Education, training, and library**	10,233	867	8.5 (7.7–9.3)	51.5 (46.9–56.2)	8.8 (6.0–11.6)	
Postsecondary teachers	1,623	113	6.9 (5.4–8.5)	38.1 (27.7–48.5)	—	
Primary, secondary, and special education school teachers	6,046	525	8.7 (7.6–9.8)	51.7 (45.7–57.7)	8.5 (4.9–12.2)	
Other teachers and instructors	1,078	82	7.7 (5.3–10.1)	48.3 (32.6–64.0)	—	
Librarians, curators, and archivists	324	—	—	—	—	
Other education, training, and library occupations	1,162	124	10.7 (8.0–13.3)	64.0 (51.7–76.4)	—	
**Arts, design, entertainment, sports, and media**	3,408	273	8.0 (6.7–9.3)	51.0 (41.7–60.3)	—	
Art and design workers	1,242	108	8.7 (6.3–11.1)	52.0 (36.9–67.0)	—	
Entertainers and performers, sports and related workers	794	64	8.0 (5.0–11.0)	—	—	
Media and communication workers	966	77	8.0 (5.7–10.3)	57.2 (42.5–71.9)	—	
Media and communication equipment workers	406	25	6.1 (2.9–9.3)	62.8 (35.7–89.8)	—	
**Office and administrative support**	19,777	1,588	8.0 (7.5–8.6)	44.8 (41.2–48.5)	8.6 (6.8–10.5)	
Supervisors, office and administrative support workers	1,256	112	8.9 (6.7–11.2)	38.3 (25.9–50.8)	—	
Communications equipment operators	88	—	—	—	—	
Financial clerks	2,926	199	6.8 (5.6–8.0)	41.1 (30.7–51.5)	6.1 (2.6–9.5)	
Information and record clerks	5,427	479	8.8 (7.7–10.0)	41.6 (35.3–47.9)	8.6 (5.3–11.9)	
Material recording, scheduling, dispatching, and distributing workers	3,993	282	7.1 (5.8–8.4)	44.6 (33.9–55.2)	6.8 (3.2–10.3)	
Secretaries and administrative assistants	2,907	209	7.2 (5.9–8.5)	46.9 (37.7–56.1)	11.9 (4.9–18.9)	
Other office and administrative support workers	3,181	281	8.9 (7.6–10.2)	54.8 (47.6–62.1)	11.4 (6.5–16.3)	
**Food preparation and serving related**	8,771	668	7.7 (6.7–8.6)	40.2 (34.2–46.1)	10.7 (7.2–14.3)	
Supervisors, food preparation, and serving workers	951	78	8.3 (5.3–11.2)	52.8 (35.0–70.7)	—	
Cooks and food preparation workers	3,317	232	7.0 (5.7–8.3)	41.2 (32.1–50.4)	12.7 (6.3–19.2)	
Food and beverage serving working	3,617	292	8.2 (6.7–9.6)	38.1 (29.3–46.9)	10.4 (5.3–15.6)	
Other food preparation and serving related workers	887	65	7.4 (5.1–9.7)	30.4 (15.1–45.7)	—	
**Community and social services**	2,862	217	7.6 (6.5–8.8)	46.0 (37.5–54.4)	6.3 (2.8–9.7)	
Counselors, social workers, and other community and social service specialists	2,199	173	7.9 (6.6–9.2)	43.0 (34.4–51.6)	7.4 (3.1–11.7)	
Religious workers	663	44	6.7 (4.2–9.1)	57.5 (38.0–77.1)	—	
**Business and financial operations**	7,710	588	7.6 (6.7–8.5)	41.0 (34.9–47.1)	9.5 (5.9–13.0)	
Business operations specialists	4,162	319	7.7 (6.5–8.8)	37.9 (30.1–45.7)	8.8 (4.2–13.3)	
Financial specialists	3,548	269	7.6 (6.2–8.9)	44.8 (35.8–53.7)	10.3 (4.9–15.7)	
**Legal**	1,791	136	7.6 (5.8–9.4)	38.6 (27.1–50.1)	—	
Lawyers, judges, and related workers	1,109	97	8.7 (6.2–11.3)	34.6 (20.7–48.5)	—	
Legal support workers	682	40	5.8 (3.6–8.1)	48.3 (29.1–67.5)	—	
**Sales and related**	16,266	1,152	7.1 (6.5–7.7)	42.9 (38.6–47.2)	12.4 (9.6–15.3)	
Supervisors, sales workers	3,985	234	5.9 (4.9–6.9)	43.7 (34.6–52.8)	—	
Retail sales workers	7,364	644	8.8 (7.8–9.7)	44.1 (38.4–49.9)	15.4 (11.2–19.6)	
Sales representatives, services	1,911	121	6.3 (4.6–8.1)	34.2 (21.6–46.7)	—	
Sales representatives, wholesale and manufacturing	1,392	61	4.4 (2.9–5.8)	41.0 (25.3–56.6)	—	
Other sales and related workers	1,614	92	5.7 (4.1–7.3)	45.3 (31.4–59.3)	—	
**Protective service**	3,272	232	7.1 (5.7–8.5)	40.4 (30.3–50.4)	—	
First–line supervisors/managers, protective service workers	211	—	—	—	—	
Firefighting and prevention workers	347	18	5.1 (2.2–8.0)	—	—	
Law enforcement workers	1,306	105	8.1 (5.3–10.9)	39.1 (22.2–55.9)	—	
Other protective service workers	1,408	95	6.8 (5.0–8.6)	40.1 (26.8–53.4)	—	
**Life, physical, and social science**	1,668	110	6.6 (4.7–8.5)	41.5 (28.0–54.9)	—	
Life scientists	348	24	6.9 (3.0–10.8)	35.7 (16.5–54.8)	—	
Physical scientists	541	28	5.2 (2.8–7.7)	47.6 (25.1–70.1)	—	
Social scientists and related workers	414	—	—	—	—	
Life, physical, and social science technicians	365	20	5.4 (2.8–8.1)	—	—	
**Management**	15,259	956	6.3 (5.7–6.8)	46.9 (42.0–51.7)	6.9 (4.4–9.4)	
Chief executives; general and operations managers; legislators	2,172	138	6.3 (5.0–7.7)	36.3 (24.6–48.1)	—	
Advertising, marketing, promotions, public relations, and sales managers	1,068	61	5.7 (3.8–7.5)	49.8(33.8–65.8)	—	
Operations specialties managers	2,911	171	5.9 (4.7–7.1)	38.8 (28.1–49.4)	—	
Other management occupations	9,108	588	6.5 (5.7–7.2)	51.4 (45.4–57.3)	7.3 (3.8–10.7)	
**Architecture and engineering**	3,301	175	5.3 (4.2–6.4)	38.4 (27.9–49.0)	—	
Architects, surveyors, and cartographers	291	—	—	—	—	
Engineers	2,272	111	4.9 (3.7–6.1)	39.9 (27.1–52.7)	—	
Drafters, engineering, and mapping technicians	738	44	5.9 (3.6–8.2)	33.6 (13.3–53.9)	—	
**Computer and mathematical**	5,021	290	5.8 (4.8–6.8)	46.3 (38.1–54.5)	—	
Computer specialists	4,774	276	5.8 (4.8–6.8)	45.9 (37.4–54.3)	—	
Mathematical science occupations	247	—	—	—	—	
**Building and grounds cleaning and maintenance**	6,518	364	5.6 (4.8–6.4)	51.0 (43.9–58.1)	15.1 (10.1–20.1)	
Supervisors, building and grounds cleaning and maintenance workers	510	30	5.8 (2.6–9.0)	55.5 (30.8–80.1)	—	
Building cleaning and pest control workers	4,552	307	6.8 (5.8–7.8)	51.7 (43.9–59.5)	16.7 (10.9–22.5)	
Grounds maintenance workers	1,456	28	1.9 (1.1–2.7)	38.9 (16.1–61.8)	—	
**Installation, maintenance, and repair**	5,513	312	5.7 (4.7–6.7)	39.1 (30.2–48.0)	—	
Supervisors of installation, maintenance, and repair workers	315	36	11.5 (5.9–17.1)	—	—	
Electrical and electronic equipment mechanics, installers, and repairers	681	33	4.8 (2.8–6.9)	—	—	
Vehicle and mobile equipment mechanics, installers, and repairers	2,076	100	4.8 (3.3–6.4)	28.7 (15.4–42.0)	—	
Other installation, maintenance, and repair occupations	2,441	143	5.9 (4.3–7.4)	46.7 (33.8–59.7)	—	
**Farming, fishing, and forestry**	1,278	68	5.4 (3.6–7.1)	43.8 (26.4–61.2)	—	
Supervisors, farming, fishing, and forestry workers	59	—	—	—	—	
Agricultural workers	1,084	67	6.2 (4.2–8.2)	43.5 (25.8–61.1)	—	
Fishing and hunting workers	45	—	—	—	—	
Forest, conservation, and logging workers	90	—	—	—	—	
**Transportation and material moving**	9,240	494	5.4 (4.7–6.1)	49.3 (42.8–55.8)	11.6 (8.3–14.9)	
Supervisors, transportation and material moving workers	171	—	—	—	—	
Air transportation workers	236	17	7.0 (3.2–10.8)	56.7 (29.7–83.7)	—	
Motor vehicle operators	4,390	211	4.8 (4.0–5.7)	45.1 (36.8–53.5)	15.5 (9.5–21.5)	
Rail transportation workers	100	—	—	—	—	
Water transportation workers	60	—	—	—	—	
Other transportation workers	327	—	—	—	—	
Material moving workers	3,957	226	5.7 (4.6–6.9)	48.9 (38.4–59.3)	9.4 (5.1–13.6)	
**Production**	9,490	484	5.1 (4.5–5.8)	36.3 (30.6–42.0)	10.2 (6.9–13.5)	
Supervisors, production workers	821	55	6.7 (3.9–9.4)	39.5 (19.0–60.1)	—	
Assemblers and fabricators	1,409	71	5.0 (3.4–6.7)	37.9 (20.4–55.5)	—	
Food processing workers	776	43	5.6 (3.4–7.7)	32.7 (15.0–50.3)	—	
Metal workers and plastic workers	1,938	115	5.9 (4.2–7.7)	30.2 (17.5–42.9)	—	
Printing workers	288	—	—	—	—	
Textile, apparel, and furnishings workers	682	26	3.9 (1.8–5.9)	37.7 (15.7–59.6)	—	
Woodworkers	155	—	—	—	—	
Plant and system operators	274	—	—	—	—	
Other production occupations	3,148	132	4.2 (3.3–5.1)	40.8 (30.0–51.6)	—	
**Construction and extraction**	8,139	324	4.0 (3.4–4.6)	37.5 (30.3–44.8)	8.2 (4.6–11.7)	
Supervisors, construction and extraction workers	649	26	4.0 (1.9–6.1)	—	—	
Construction trades workers	6,789	264	3.9 (3.2–4.6)	35.4 (27.7–43.0)	8.8 (4.7–12.9)	
Helpers, construction trades	59	—	—	—	—	
Other construction and related workers	402	—	—	—	—	
Extraction workers	240	—	—	—	—	
**Military**	367	—	—	—	—	
Refused, not ascertained, don’t know	2,653	92	3.5 (2.5–4.5)	50.1 (36.2–64.1)	—	
**Total**	**160,672**	**10,957**	**6.8 (6.7–7.0)**	**44.7 (43.3–46.1)**	**9.9 (9.1–10.7)**	

**Abbreviation:** CI = confidence interval.

* Defined as a “yes” response to the questions “Have you ever been told by a doctor or other health professional that you had asthma?” and “Do you still have asthma?”

^†^ Survey respondents who answered “yes” to the question “Did you have a job or business at any time in the past 12 months?”

^§^ Defined as a “yes” response to the question “During the past 12 months, have you had an episode of asthma or an asthma attack?”

^¶^ Defined as a “yes” response to the question “During the past 12 months, have you had to visit an emergency room or urgent care center because of asthma?”

** Occupation that employed sample adults had during the week prior to their interview. ftp://ftp.cdc.gov/pub/Health_Statistics/NCHS/Dataset_Documentation/NHIS/2014/srvydesc.pdf.

^††^ Weighted to provide national estimates.

^§§^ Estimates suppressed because relative standard error for the estimate was ≥30%.

By detailed occupation subgroup, the highest prevalence of current asthma (10.7%) and asthma attack in the past 12 months (64.0%) was among workers in other education, training, and library occupations^††^ (Table 3). Prevalence of asthma-related ED visits was highest among personal appearance workers^§§^ (25.0%). The highest number of workers with asthma attacks was among those working in other management occupations^¶¶^ (302,000), and the highest number of workers with asthma-related ED visits was among retail sales workers (99,000).

## Discussion

This report provides industry- and occupation-specific prevalence estimates of current asthma, and among those with current asthma, the prevalence of at least one asthma attack and at least one asthma-related ED visit in the past year. The numbers of workers reporting asthma attacks and asthma-related ED visits in specific industries and occupations correlate with the numbers of workers and current asthma prevalence in each group. The increased prevalence of current asthma, asthma attacks, and asthma-related ED visits in certain industries and occupations might indicate increased risks for these health outcomes associated with workplace exposures. The highest prevalence of current asthma was among workers in the health care and social assistance industry and in health care support occupations. New-onset work-related asthma in these workers has been associated with exposure to cleaning and disinfecting products, powdered latex gloves, and aerosolized medications (*4*). Nearly two thirds of the workers with asthma in the wood products and in the plastics and rubber products manufacturing industries had at least one asthma attack in the past year. Workers in these industries are at increased risk for work-related asthma (*5*,*6*), and the high proportion of workers with a history of an asthma attack in this report suggests a high risk for work-related exacerbation of asthma. Education, training, and library workers are also at risk for work-related asthma and adverse health outcomes (*7*).

NHIS did not collect data on severity of asthma exacerbations and asthma work-relatedness. The subset of patients who experience severe asthma exacerbations have an accelerated decline in lung function, greater health care utilization, and a lower quality of life (*3*,*8*). Based on the estimate that approximately 51% of adult asthma might be caused or made worse by work (*9*), as many as 5.6 million workers might have asthma or asthma outcomes related to work that could be prevented. Physicians should consider work-related asthma in all workers with new-onset or worsening asthma (*2*,*3*).

Workplace conditions and exposures associated with asthma include irritant chemicals, dusts, secondhand tobacco smoke, allergens and sensitizers, emotional stress, worksite temperature, and physical exertion (*3*). A list of asthmagens causing work-related asthma by sensitization or acute irritant-induced asthma is available (http://www.aoecdata.org/ExpCodeLookup.aspx). Identification of potential asthma-related agents in the workplace can be facilitated by obtaining safety data sheets.*** Guidelines intended to promote effective management of work-related asthma are available (*2*,*3*). The preferred primary strategy to prevent work-related asthma and reduce signs, symptoms, and progression of disease is exposure control (i.e., elimination or substitution of hazardous products, engineering controls, and respiratory protection). However, if these approaches are unsuccessful, removal of the worker from exposure might sometimes be necessary for management of work-related asthma (*2*,*3*,*10*).

The findings in this report are subject to at least four limitations. First, information on asthma, asthma attacks, and asthma-related ED visits was self-reported and not validated by medical records. It is likely that some respondents had misdiagnosed or undiagnosed asthma. Second, no temporal information on asthma onset and exacerbations was available; thus, it was not possible to determine asthma association with work. Third, only workers employed at some time in the past 12 months were included in this study. Those with severe asthma might have left employment in industries and occupations with workplace exposures that exacerbate their asthma; thus, industry and occupation in this report might not accurately identify workers’ industry and occupation where exposures occur. Finally, small sample sizes for some groups resulted in unreliable estimates.

These findings might assist physicians to identify workers who should be evaluated for possible work-related asthma in industries and occupations with a high prevalence of asthma, asthma attacks, and asthma-related ED visits and could help public health officials identify workplaces where detailed investigations for prevention and control might be appropriate. Continued surveillance is important to assess asthma prevalence and trends by respondents’ industry and occupation.

**Summary**

**What is already known about this topic?**

In 2010, an estimated 8.2% of U.S. adults had current asthma; among them, 49.1% reported at least one asthma attack in the past year. Up to 51% of adult asthma might be related to work and could therefore potentially be prevented.

**What is added by this report?**

During 2011–2016, among an estimated 160.7 million working adults, 6.8% had current asthma. Among those with asthma, 44.7% experienced an asthma attack, and 9.9% had an asthma-related emergency department visit in the previous year. The current asthma prevalence was highest among workers employed in the health care and social assistance industry (8.8%) and in health care support occupations (8.8%).

**What are the implications for public health practice?**

This information might assist physicians to identify workers who should be evaluated for possible work-related asthma and could help public health officials identify workplaces where detailed investigations for prevention and control might be appropriate. Guidelines promoting effective management of work-related asthma are available.

## Footnotes

* Work-related asthma is defined as either occupational asthma (i.e., new-onset asthma caused by factors related to work) or work-exacerbated asthma (i.e., preexisting or concurrent asthma worsened by factors related to work).

† https://www.cdc.gov/nchs/nhis/quest_data_related_1997_forward.htm.

§ ftp://ftp.cdc.gov/pub/Health_Statistics/NCHS/Dataset_Documentation/NHIS/2014/samadult_layout.pdf.

¶ Industry and occupation information that employed sample adults had during the week before the interview. Additional information on the industry and occupation coding schemes can be found at ftp://ftp.cdc.gov/pub/Health_Statistics/NCHS/Dataset_Documentation/NHIS/2014/srvydesc.pdf.

** Poverty index is based on family income and family size using the U.S. Census Bureau’s poverty thresholds for the previous calendar year. Persons categorized as “poor” have family incomes <100% of the poverty threshold, “near poor” have family incomes ≥100% to <200% of the poverty threshold, “not poor” have family incomes ≥200% of the poverty threshold. ftp://ftp.cdc.gov/pub/Health_Statistics/NCHS/Dataset_Documentation/NHIS/2016/srvydesc.pdf, https://www.census.gov/data/tables/time-series/demo/income-poverty/historical-poverty-thresholds.html.

†† Audio-visual and multimedia collections specialists; farm and home management advisors; instructional coordinators; teacher assistants; miscellaneous education, training, and library workers (25-9000 Other Education, Training, and Library Occupations). https://www.bls.gov/soc/2010/2010_major_groups.htm#25-0000.

§§ Barbers; hairdressers, hairstylists and cosmetologists; makeup artists, theatrical and performance; manicurists and pedicurists; shampooers; skin care specialists (39-5000 Personal Appearance Workers). https://www.bls.gov/soc/2010/2010_major_groups.htm#39-0000.

¶¶ Farmers, ranchers, and other agricultural managers; construction managers; education administrators; architectural and engineering managers; food service managers; funeral service managers; gaming managers; lodging managers; medical and health services managers; natural sciences managers; postmasters and mail superintendents; property, real estate, and community association managers; social and community service managers; emergency management directors (11-9000 Other Management Occupations). https://www.bls.gov/soc/2010/2010_major_groups.htm#11-0000.

*** https://www.osha.gov/Publications/HazComm_QuickCard_SafetyData.html.
